# Supplementary material for: Proteomic Analysis of Differentially Expressed Proteins in A549 Cells Infected with H9N2 Avian Influenza Virus
Source: Int J Mol Sci. 2025 Jan 14;26(2):657. doi: 10.3390/ijms26020657 (PMC11765812; doi:10.3390/ijms26020657)
Supplement: Supplementary file 1 [file ijms-26-00657-s001.zip › ijms-3370944-supplementary.pdf]

Supplementary Table S1 Top 10 regulated DEPs in our proteomic data.

Protein ID	Protein name	Protein description	Regulated type
P62633	CNBP	Cellular nucleic acid-binding protein	Up
Q3ZAQ7	VMA21	Vacuolar ATPase assembly integral membrane protein	Up
Q16799	RTN1	Reticulon-1	Up
Q15646	OASL	2'-5'-oligoadenylate synthase-like protein	Up
Q9UK76	JPT1	Jupiter microtubule associated homolog 1	Up
Q02297	NRG1	Pro-neuregulin-1, membrane-bound isoform	Down
Q9H694	BICC1	Protein bicaudal C homolog 1	Down
Q15043	SLC39A14	Metal cation symporter ZIP14	Down
Q8IXZ2	ZC3H3	Zinc finger CCCH domain-containing protein 3	Down
P40763	STAT3	Signal transducer and activator of transcription 3	Down
